# *N*,*N*′-(Propane-1,3-di­yl)dibenzo­thio­amide

**DOI:** 10.1107/S160053681400974X

**Published:** 2014-05-03

**Authors:** Masayuki Nagasawa, Yuji Sasanuma, Hyuma Masu

**Affiliations:** a Department of Applied Chemistry and Biotechnology, Chiba University, 1-33 Yayoi-cho, Inage-ku, Chiba 263-8522, Japan; b Center for Analytical Instrumentation, Chiba University, 1-33 Yayoi-cho, Inage-ku, Chiba 263-8522, Japan

## Abstract

The title compound, C_17_H_18_N_2_S_2_, exhibits a *trans*–*trans*–*trans*–*gauche^+^* (*tttg* ^+^) conformation with regard to the NH–CH_2_–CH_2_–CH_2_–NH bond sequence. In the crystal, mol­ecules are connected by N—H⋯S=C and C—H⋯S=C hydrogen bonds, forming a herringbone arrangement along the *c*-axis direction. The two thioamide groups make dihedral angles of 43.0 (2) and 33.1 (2)° with the adjacent phenyl rings.

## Related literature   

For the crystal structures and conformations of related compounds, see: for example, Palmer & Brisse (1980 ▶); Brisson & Brisse (1986 ▶); Deguire & Brisse (1988 ▶); Nagasawa *et al.* (2014 ▶). For the synthesis, see: Hart & Brewbaker (1969 ▶); Cave & Levinson (1985 ▶).
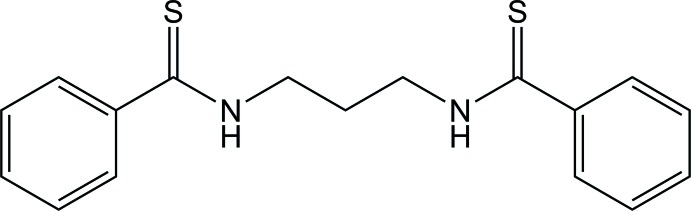


## Experimental   

### 

#### Crystal data  

C_17_H_18_N_2_S_2_
*M* *_r_* = 314.45
Orthorhombic, 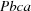

*a* = 8.36521 (9) Å
*b* = 14.13395 (14) Å
*c* = 26.9223 (3) Å
*V* = 3183.12 (6) Å^3^
*Z* = 8
Cu *K*α radiation
μ = 2.97 mm^−1^
*T* = 223 K
0.30 × 0.20 × 0.10 mm

#### Data collection  

Bruker APEXII Ultra CCD area-detector diffractometer
Absorption correction: multi-scan (*SADABS*; Bruker, 2001 ▶) *T*_min_ = 0.47, *T*_max_ = 0.76
12276 measured reflections
2882 independent reflections
2736 reflections with *I* > 2σ(*I*)
*R*_int_ = 0.018

#### Refinement  

*R*[*F* ^2^ > 2σ(*F* ^2^)] = 0.032
*wR*(*F* ^2^) = 0.085
*S* = 1.05
2882 reflections
190 parameters
H-atom parameters constrained
Δρ_max_ = 0.26 e Å^−3^
Δρ_min_ = −0.31 e Å^−3^

### 

Data collection: *APEX2* (Bruker, 2007 ▶); cell refinement: *SAINT* (Bruker, 2007 ▶); data reduction: *SAINT*; program(s) used to solve structure: *SHELXS2013* (Sheldrick, 2008 ▶); program(s) used to refine structure: *SHELXL2013* (Sheldrick, 2008 ▶); molecular graphics: *Mercury* (Macrae *et al.*, 2006 ▶); software used to prepare material for publication: *SHELXL2013*.

## Supplementary Material

Crystal structure: contains datablock(s) global, I. DOI: 10.1107/S160053681400974X/bv2233sup1.cif

Structure factors: contains datablock(s) I. DOI: 10.1107/S160053681400974X/bv2233Isup2.hkl

Supporting information file. DOI: 10.1107/S160053681400974X/bv2233Isup3.cml

CCDC reference: 1000286

Additional supporting information:  crystallographic information; 3D view; checkCIF report

## Figures and Tables

**Table 1:** Hydrogen-bond geometry (Å, °)

*D*—H⋯*A*	*D*—H	H⋯*A*	*D*⋯*A*	*D*—H⋯*A*
N1—H1⋯S2^i^	0.87	2.59	3.4412 (14)	168
N2—H2⋯S1^ii^	0.87	2.69	3.5135 (12)	157
C17—H17⋯S1^iii^	0.94	2.85	3.7665 (17)	166

Symmetry codes: (i) 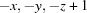; (ii) 
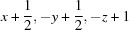
; (iii) 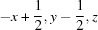.

**Fig. 1.**
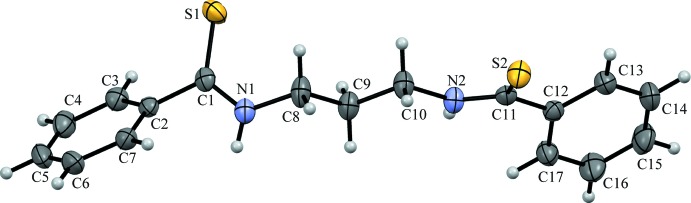
Molecular structure of N, N'-(propane-1,2-diyl)dibenzothioamide (PDBTA). Displacement ellipsoids are drawn at the 50% probability level. Isotropic H-atom thermal parameters are represented by spheres of arbitrary size. The labels of hydrogen atoms are omitted for clarity.

**Fig. 2.**
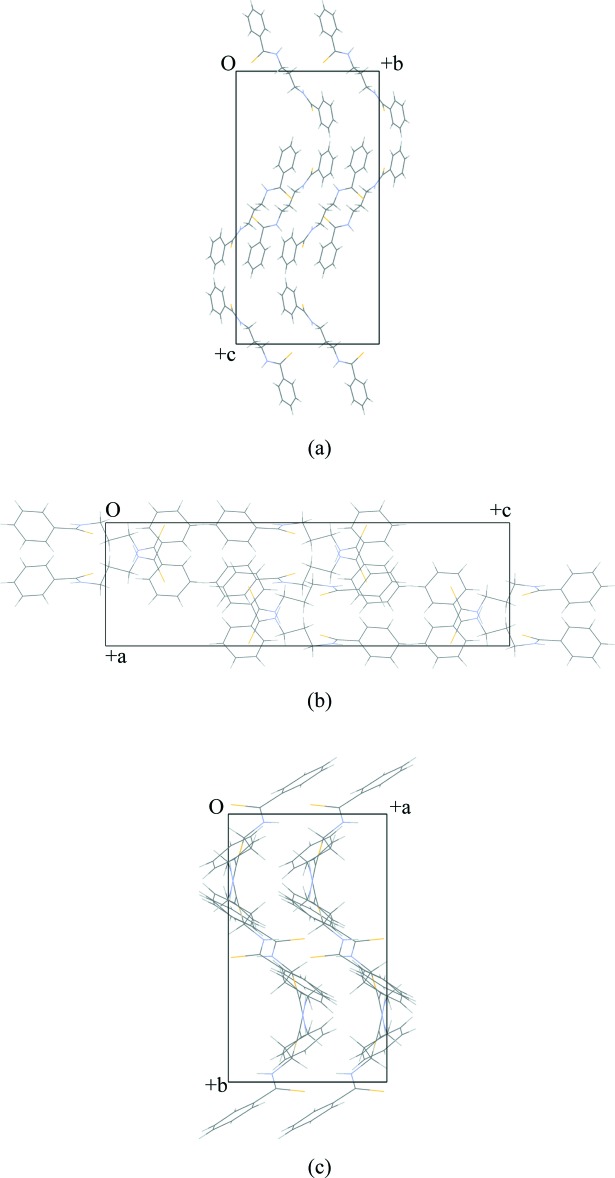
Packing diagram of PDBTA, viewed down the (a) a, (b) b, and (c) c axes.

## supplementary crystallographic information

### 1. Comment

In a previous paper (Nagasawa *et al.*, 2014), we reported the crystal structure of *N, N'*-(ethane-1,2-diyl)dibenzothioamide. In this study, we have determined the crystal structure of its homologue, *N,N'*-(propane-1,3-diyl)dibenzothioamide (referred to here as PDBTA), a monomeric model compound of poly(trimethylene terephthalthioamide), [–(C=S)–C_6_H_4_–(C=S)–NH–(CH_2_)_3_–NH–]*_n_*. Figure 1 shows the molecular structure of PDBTA, whose NH–CH_2_–CH_2_–CH_2_–NH bond sequence adopts the *tttg^+^* conformation. On the other hand, our molecular orbital (MO) calculations at the B3LYP/6–311+G(2 d,p)//B3LYP/6–311+G(2 d,p) level including the solvent effect of dimethyl sulfoxide have predicted that the all-*trans* form is the most stable of its possible conformations; the crystal conformation, *tttg^+^*, was suggested to have a free energy higher than *tttt* by as much as 1.19 kcal mol^-1^.

Figure 2 shows the molecular packing of the PDBTA crystal, in which two kinds of intermolecular hydrogen bonds, C=S···H–N and C=S···H–C, seem to be formed (not shown in the figure). For details, see Table 1. The intermolecular attractions may fully compensate the cost of conformational energy of 1.19 kcal mol^-1^. The PDBTA molecules form a herringbone arrangement along the *c*-axis direction.

Brisson & Brisse (1986) determined the crystal structure of *N,N'*-(propane-1,3-diyl)dibenzamide (PDBA), a model compound of poly(trimethylene terephthalamide). The crystalline PDBA molecule lies in the *ttg^+^g^+^* conformation and forms intermolecular C=O···H—N hydrogen bonds with the neighbors. According to our MO calculations, its most stable conformer is *tttg^+^* (–0.54 kcal mol^-1^ relative to that of the all-*trans* state), and the crystal conformation, *ttg^+^g^+^*, has a somewhat higher free energy (+0.32 kcal mol^-1^).

### 2. Experimental

Benzoyl chloride (12.0 ml, 103 mmol) was added dropwise to an aqueous solution of 1,3-diaminopropane (3.4 ml, 41 mmol) and sodium hydroxide (61.5 ml, 0.100 mol l^-1^) stirred by a mechanical stirrer in a three-necked flask equipped with a dropping funnel and a calcium-chloride drying tube, with the flask being bathed in water kept at 10 °C. The mixture was stirred at 10 °C for 1 h, diluted with water (100 ml), and stirred again at room temperature overnight to yield white precipitate. The precipitate was collected by suction filtration, washed with water, and dried. The crude product was recrystallized from a mixture of ethanol and toluene (1:1 in volume) and dried at 40 °C under reduced pressure to yield PDBA (yield 28%). In principle, this synthesis is based on the procedure of Hart & Brewbaker (1969).

PDBA (1.0 g, 3.6 mmol) and Lawesson's reagent (1.7 g, 4.2 mmol) (Cave & Levinson, 1985) were dissolved in toluene (20 ml) stirred in a three-necked flask equipped with a reflux condenser connected to a calcium-chloride drying tube. The mixture was refluxed under dry nitrogen at *ca* 110 °C for 8 h, and the completion of the reaction was confirmed by thin-layer chromatography. After toluene was removed under reduced pressure, the residual was subjected to column chromatography on silica gel with chloroform, and yellowish solution (retention factor *R_f_* = 0.3) was collected and condensed to yield yellow slurry, which was recrystallized twice from a mixture of ethyl acetate and diethyl ether (1:1 in volume) and dried under reduced pressure to yield PDBTA (yield 48%).

A small quantity of PDBTA was dissolved in chloroform in a glass tube, whose top was sealed with a thin Teflon film. The tube was placed in a vial container including a small amount of *n*-hexane, and the container was capped and left to stand still in a dark place. After a day, crystals were found to be formed suitable for X-ray diffraction.

### 3. Refinement

All C—H hydrogen atoms were geometrically positioned with C—H = 0.95 and 0.99 Å for the aromatic and methylene groups respectively, and refined as riding by *U*iso(H) = 1.2 *U*eq(C). The N—H hydrogen atoms were located and fixed with N—H = 0.87 Å.

### Crystal data

**Table:**

C_17_H_18_N_2_S_2_	*F*(000) = 1328
*M**_r_* = 314.45	*D*_x_ = 1.312 Mg m^−^^3^
Orthorhombic, *P**b**c**a*	Cu *K*α radiation, λ = 1.54178 Å
*a* = 8.36521 (9) Å	µ = 2.97 mm^−^^1^
*b* = 14.13395 (14) Å	*T* = 223 K
*c* = 26.9223 (3) Å	Prismatic, yellow
*V* = 3183.12 (6) Å^3^	0.30 × 0.20 × 0.10 mm
*Z* = 8	

### Data collection

**Table:**

Bruker APEXII Ultra CCD area-detector diffractometer	2882 independent reflections
Radiation source: Bruker TXS fine-focus rotating anode	2736 reflections with *I* > 2σ(*I*)
Bruker Helios multilayer mirror monochromator	*R*_int_ = 0.018
Phi and ω scans	θ_max_ = 68.3°, θ_min_ = 3.3°
Absorption correction: multi-scan (*SADABS*; Bruker, 2001)	*h* = −9→10
*T*_min_ = 0.47, *T*_max_ = 0.76	*k* = −16→17
12276 measured reflections	*l* = −32→32

### Refinement

**Table:**

Refinement on *F*^2^	Primary atom site location: structure-invariant direct methods
Least-squares matrix: full	Secondary atom site location: difference Fourier map
*R*[*F*^2^ > 2σ(*F*^2^)] = 0.032	Hydrogen site location: inferred from neighbouring sites
*wR*(*F*^2^) = 0.085	H-atom parameters constrained
*S* = 1.05	*w* = 1/[σ^2^(*F*_o_^2^) + (0.0498*P*)^2^ + 1.0464*P*] where *P* = (*F*_o_^2^ + 2*F*_c_^2^)/3
2882 reflections	(Δ/σ)_max_ = 0.001
190 parameters	Δρ_max_ = 0.26 e Å^−^^3^
0 restraints	Δρ_min_ = −0.31 e Å^−^^3^

### Special details

**Table:**

Experimental. *SADABS* (Sheldrick 1996)
Geometry. All e.s.d.'s (except the e.s.d. in the dihedral angle between two l.s. planes) are estimated using the full covariance matrix. The cell e.s.d.'s are taken into account individually in the estimation of e.s.d.'s in distances, angles and torsion angles; correlations between e.s.d.'s in cell parameters are only used when they are defined by crystal symmetry. An approximate (isotropic) treatment of cell e.s.d.'s is used for estimating e.s.d.'s involving l.s. planes.
Refinement. Refinement of *F*^2^ was performed with all reflections. The weighted *R*-factor (*wR*) and goodness of fit (*S*) are based on *F*^2^, while the *R*-factor on *F*. The threshold expression of *F*^2^ > 2.0 σ(*F*^2^) was used only for calculating *R*-factor.

### Fractional atomic coordinates and isotropic or equivalent isotropic displacement parameters (Å^2^)

**Table:**

	*x*	*y*	*z*	*U*_iso_*/*U*_eq_	
C1	0.04995 (15)	0.30766 (10)	0.42684 (5)	0.0271 (3)	
C2	0.04043 (16)	0.33224 (9)	0.37326 (5)	0.0261 (3)	
C3	0.15288 (18)	0.39267 (10)	0.35235 (5)	0.0328 (3)	
H3	0.236	0.4175	0.3719	0.039*	
C4	0.1418 (2)	0.41607 (11)	0.30247 (6)	0.0408 (4)	
H4	0.2193	0.4555	0.288	0.049*	
C5	0.0177 (2)	0.38189 (12)	0.27379 (6)	0.0435 (4)	
H5	0.0109	0.3981	0.24	0.052*	
C6	−0.0964 (2)	0.32377 (11)	0.29487 (6)	0.0400 (4)	
H6	−0.1823	0.3017	0.2756	0.048*	
C7	−0.08486 (17)	0.29786 (10)	0.34432 (5)	0.0318 (3)	
H7	−0.1614	0.2572	0.3584	0.038*	
C8	−0.00172 (17)	0.17921 (12)	0.48748 (6)	0.0380 (4)	
H8A	−0.092	0.1351	0.487	0.046*	
H8B	−0.0292	0.231	0.5101	0.046*	
C9	0.14371 (17)	0.12807 (10)	0.50784 (5)	0.0340 (3)	
H9A	0.2339	0.172	0.5108	0.041*	
H9B	0.1747	0.0765	0.4855	0.041*	
C10	0.09938 (18)	0.08896 (11)	0.55852 (6)	0.0364 (3)	
H10A	0.0758	0.1419	0.5809	0.044*	
H10B	0.0019	0.051	0.5553	0.044*	
C11	0.19903 (16)	−0.02401 (9)	0.61993 (5)	0.0268 (3)	
C12	0.33873 (16)	−0.07725 (9)	0.63952 (5)	0.0270 (3)	
C13	0.34922 (18)	−0.09590 (10)	0.69027 (5)	0.0330 (3)	
H13	0.2681	−0.0749	0.7118	0.04*	
C14	0.4785 (2)	−0.14514 (12)	0.70912 (6)	0.0417 (4)	
H14	0.4858	−0.1563	0.7435	0.05*	
C15	0.5967 (2)	−0.17792 (12)	0.67788 (7)	0.0478 (4)	
H15	0.6842	−0.2117	0.6908	0.057*	
C16	0.5859 (2)	−0.16086 (13)	0.62749 (7)	0.0480 (4)	
H16	0.6657	−0.1839	0.6061	0.058*	
C17	0.45871 (19)	−0.11019 (11)	0.60823 (6)	0.0368 (3)	
H17	0.4534	−0.098	0.5739	0.044*	
N1	0.01881 (14)	0.21803 (9)	0.43765 (5)	0.0324 (3)	
H1	0.0098	0.1789	0.4129	0.039*	
N2	0.22457 (14)	0.03070 (8)	0.58067 (4)	0.0301 (3)	
H2	0.3197	0.0319	0.5676	0.036*	
S1	0.08969 (5)	0.39123 (3)	0.46914 (2)	0.03743 (13)	
S2	0.01763 (4)	−0.03524 (3)	0.64602 (2)	0.03725 (13)	

### Atomic displacement parameters (Å^2^)

**Table:**

	*U*^11^	*U*^22^	*U*^33^	*U*^12^	*U*^13^	*U*^23^
C1	0.0195 (6)	0.0338 (7)	0.0279 (7)	0.0044 (5)	−0.0002 (5)	0.0037 (5)
C2	0.0273 (6)	0.0256 (6)	0.0253 (6)	0.0051 (5)	0.0003 (5)	0.0011 (5)
C3	0.0312 (7)	0.0339 (7)	0.0333 (8)	0.0012 (6)	0.0037 (6)	0.0018 (5)
C4	0.0464 (9)	0.0401 (8)	0.0360 (8)	0.0058 (7)	0.0134 (7)	0.0093 (6)
C5	0.0620 (11)	0.0451 (9)	0.0235 (7)	0.0163 (8)	0.0029 (7)	0.0045 (6)
C6	0.0516 (9)	0.0382 (8)	0.0301 (7)	0.0063 (7)	−0.0113 (6)	−0.0039 (6)
C7	0.0360 (8)	0.0273 (6)	0.0320 (7)	0.0012 (5)	−0.0039 (6)	0.0008 (5)
C8	0.0310 (7)	0.0457 (9)	0.0372 (8)	0.0039 (6)	0.0028 (6)	0.0188 (7)
C9	0.0303 (7)	0.0361 (7)	0.0356 (8)	0.0006 (6)	−0.0008 (6)	0.0106 (6)
C10	0.0330 (8)	0.0413 (8)	0.0349 (8)	0.0058 (6)	−0.0007 (6)	0.0122 (6)
C11	0.0315 (7)	0.0231 (6)	0.0259 (6)	−0.0012 (5)	−0.0023 (5)	−0.0014 (5)
C12	0.0296 (7)	0.0233 (6)	0.0281 (6)	−0.0012 (5)	−0.0016 (5)	0.0010 (5)
C13	0.0391 (8)	0.0325 (7)	0.0275 (7)	0.0016 (6)	0.0002 (6)	0.0016 (5)
C14	0.0506 (9)	0.0427 (8)	0.0317 (8)	0.0055 (7)	−0.0086 (7)	0.0083 (7)
C15	0.0453 (9)	0.0478 (9)	0.0502 (10)	0.0152 (7)	−0.0079 (7)	0.0108 (8)
C16	0.0438 (9)	0.0545 (10)	0.0456 (9)	0.0193 (8)	0.0080 (7)	0.0056 (8)
C17	0.0412 (8)	0.0414 (8)	0.0279 (7)	0.0074 (7)	0.0028 (6)	0.0045 (6)
N1	0.0338 (6)	0.0338 (6)	0.0298 (6)	0.0025 (5)	−0.0009 (5)	0.0071 (5)
N2	0.0266 (6)	0.0323 (6)	0.0315 (6)	0.0001 (4)	−0.0015 (5)	0.0075 (5)
S1	0.0403 (2)	0.0455 (2)	0.0265 (2)	0.00085 (15)	−0.00578 (14)	−0.00393 (14)
S2	0.0315 (2)	0.0413 (2)	0.0390 (2)	0.00656 (14)	0.00747 (14)	0.00916 (15)

### Geometric parameters (Å, º)

**Table:**

C1—N1	1.3256 (18)	C9—H9B	0.98
C1—C2	1.4860 (17)	C10—N2	1.4596 (18)
C1—S1	1.6741 (14)	C10—H10A	0.98
C2—C3	1.390 (2)	C10—H10B	0.98
C2—C7	1.393 (2)	C11—N2	1.3270 (17)
C3—C4	1.386 (2)	C11—C12	1.4867 (18)
C3—H3	0.94	C11—S2	1.6795 (14)
C4—C5	1.381 (3)	C12—C17	1.391 (2)
C4—H4	0.94	C12—C13	1.3942 (19)
C5—C6	1.381 (2)	C13—C14	1.382 (2)
C5—H5	0.94	C13—H13	0.94
C6—C7	1.384 (2)	C14—C15	1.379 (2)
C6—H6	0.94	C14—H14	0.94
C7—H7	0.94	C15—C16	1.381 (2)
C8—N1	1.4595 (18)	C15—H15	0.94
C8—C9	1.5175 (19)	C16—C17	1.383 (2)
C8—H8A	0.98	C16—H16	0.94
C8—H8B	0.98	C17—H17	0.94
C9—C10	1.518 (2)	N1—H1	0.87
C9—H9A	0.98	N2—H2	0.87
			
N1—C1—C2	115.21 (12)	N2—C10—C9	113.41 (12)
N1—C1—S1	124.33 (11)	N2—C10—H10A	108.9
C2—C1—S1	120.40 (10)	C9—C10—H10A	108.9
C3—C2—C7	119.80 (13)	N2—C10—H10B	108.9
C3—C2—C1	120.04 (12)	C9—C10—H10B	108.9
C7—C2—C1	120.11 (12)	H10A—C10—H10B	107.7
C4—C3—C2	119.58 (14)	N2—C11—C12	116.80 (12)
C4—C3—H3	120.2	N2—C11—S2	122.25 (10)
C2—C3—H3	120.2	C12—C11—S2	120.93 (10)
C5—C4—C3	120.58 (15)	C17—C12—C13	119.01 (13)
C5—C4—H4	119.7	C17—C12—C11	121.46 (12)
C3—C4—H4	119.7	C13—C12—C11	119.52 (12)
C4—C5—C6	119.84 (14)	C14—C13—C12	120.27 (14)
C4—C5—H5	120.1	C14—C13—H13	119.9
C6—C5—H5	120.1	C12—C13—H13	119.9
C5—C6—C7	120.29 (14)	C15—C14—C13	120.44 (14)
C5—C6—H6	119.9	C15—C14—H14	119.8
C7—C6—H6	119.9	C13—C14—H14	119.8
C6—C7—C2	119.87 (14)	C14—C15—C16	119.57 (14)
C6—C7—H7	120.1	C14—C15—H15	120.2
C2—C7—H7	120.1	C16—C15—H15	120.2
N1—C8—C9	114.63 (12)	C15—C16—C17	120.60 (15)
N1—C8—H8A	108.6	C15—C16—H16	119.7
C9—C8—H8A	108.6	C17—C16—H16	119.7
N1—C8—H8B	108.6	C16—C17—C12	120.09 (14)
C9—C8—H8B	108.6	C16—C17—H17	120.0
H8A—C8—H8B	107.6	C12—C17—H17	120.0
C8—C9—C10	107.59 (12)	C1—N1—C8	125.74 (13)
C8—C9—H9A	110.2	C1—N1—H1	117.1
C10—C9—H9A	110.2	C8—N1—H1	117.1
C8—C9—H9B	110.2	C11—N2—C10	122.59 (12)
C10—C9—H9B	110.2	C11—N2—H2	118.7
H9A—C9—H9B	108.5	C10—N2—H2	118.7

### Hydrogen-bond geometry (Å, º)

**Table:**

*D*—H···*A*	*D*—H	H···*A*	*D*···*A*	*D*—H···*A*
N1—H1···S2^i^	0.87	2.59	3.4412 (14)	168
N2—H2···S1^ii^	0.87	2.69	3.5135 (12)	157
C17—H17···S1^iii^	0.94	2.85	3.7665 (17)	166

Symmetry codes: (i) −*x*, −*y*, −*z*+1; (ii) *x*+1/2, −*y*+1/2, −*z*+1; (iii) −*x*+1/2, *y*−1/2, *z*.
